# Defective Cystinosin, Aberrant Autophagy−Endolysosome Pathways, and Storage Disease: Towards Assembling the Puzzle

**DOI:** 10.3390/cells11030326

**Published:** 2022-01-19

**Authors:** Laura Rita Rega, Ester De Leo, Daniela Nieri, Alessandro Luciani

**Affiliations:** 1 Renal Diseases Research Unit, Genetic and Rare Diseases Research Area, Bambino Gesù Children’s Hospital, IRCCS, 00146 Rome, Italy; ester.deleo@opbg.net; 2 Mechanisms of Inherited Kidney Diseases Group, Institute of Physiology, University of Zurich, 8057 Zurich, Switzerland; daniela.nieri@uzh.ch

**Keywords:** autophagy, endolysosome, epithelial cell differentiation, homeostasis, lysosomal storage diseases, mitochondrial distress, kidney proximal tubule

## Abstract

Epithelial cells that form the kidney proximal tubule (PT) rely on an intertwined ecosystem of vesicular membrane trafficking pathways to ensure the reabsorption of essential nutrients—a key requisite for homeostasis. The endolysosome stands at the crossroads of this sophisticated network, internalizing molecules through endocytosis, sorting receptors and nutrient transporters, maintaining cellular quality control via autophagy, and toggling the balance between PT differentiation and cell proliferation. Dysregulation of such endolysosome-guided trafficking pathways might thus lead to a generalized dysfunction of PT cells, often causing chronic kidney disease and life-threatening complications. In this review, we highlight the biological functions of endolysosome-residing proteins from the perspectives of understanding—and potentially reversing—the pathophysiology of rare inherited diseases affecting the kidney PT. Using cystinosis as a paradigm of endolysosome disease causing PT dysfunction, we discuss how the endolysosome governs the homeostasis of specialized epithelial cells. This review also provides a critical analysis of the molecular mechanisms through which defects in autophagy pathways can contribute to PT dysfunction, and proposes potential interventions for affected tissues. These insights might ultimately accelerate the discovery and development of new therapeutics, not only for cystinosis, but also for other currently intractable endolysosome-related diseases, eventually transforming our ability to regulate homeostasis and health.

## 1. Introduction

Epithelial cells that line the proximal tubule (PT) of the kidney reabsorb a large variety of filtered macromolecules and low-molecular mass-nutrients through a particularly well-developed endolysosome system and through membrane trafficking pathways. Ever since its discovery by Christian De Duve in the 1960s, the endolysosome has come to be known as a single-membrane-enclosed organelle devoted to the degradation of damaged cellular constituents, including aged and/or misfolded proteins, and pathogens [1]. Extracellular and intracellular materials can reach the endolysosome through endocytosis and autophagy, respectively [2]. Fusion events subsequently enable the endolysosomes to recycle cargoes and/or substrates engulfed by endocytic and/or autophagic vesicles.

Beyond degradation and the disposal of cellular waste [3], the endolysosomes can also steer the metabolic trajectory of cells in response to nutrient availability, growth factors, and stress signals, hence guiding nearly every aspect of metabolic function, ultimately coordinating cell- and organism-wide growth [4]. As a consequence, dysregulation of endolysosomes and autophagy pathways might thus pose a devastating threat to many different cell types, eventually culminating in neurodegeneration, metabolic disease, cancer, and pathologies associated with ageing [2].

Over the last two decades, studies of rare inherited diseases, in combination with advances in technology and high-throughput omics, have provided novel insights into fundamental principles governing the contribution of the endolysosome to the maintenance of homeostasis [5,6]. Through converging approaches, these paradigms have helped to understand the pathogenesis of common kidney disease entities in which imbalances in the endolysosome system have been implicated in PT dysfunction [7]. Furthermore, the integration of genome-wide association studies (GWAS) with quantitative trait analyses (eQTLs) have identified that the expression of an endolysosome-residing enzyme, that is the beta-mannosidase (MANBA), is lower in the kidneys of subjects with chronic kidney disease (CKD), and that the MANBA risk allele shows evidence of structural and functional defects in endolysosome and autophagy pathways in kidney tubule cells [8]. In addition, common variants within genes closely linked to endolysosome function (e.g., *CUBN*, *DAB2*, *RAB4*, and *LRP2*) have recently been associated with proteinuria and CKD risk [9,10], highlighting the fundamental role of the endolysosome system in kidney health and its contribution to kidney disease risk within the population.

In this review, we discuss the biological functions of the endolysosome, not only as a “disposal-garbage system” of the cell, but also as a hub for homeostasis and signaling pathways, and delve into the breakdown and removal of damaged and/or dysfunctional mitochondria through autophagic pathways. Taking cystinosis as a paradigm of endolysosome disease causing PT dysfunction, we discuss how the endolysosome and autophagy govern the physiology of kidney tubule cells. We describe the cellular pathways and molecular underpinnings through which the absence of the endolysosome-residing transporter cystinosin might wreak havoc on autophagy, ultimately leading to dysfunction of the kidney PT. In the concluding section, we highlight potential new, attractive targets for therapeutically tackling cellular adversities linked to cystinosin dysfunction, offering new targetable pathways for this life-threatening disease.

## 2. Role of the Endolysosome System and Autophagy in Kidney Proximal Tubule

### 2.1. Receptor-Mediated Endocytosis and the Endolysosome

**Figure 1:**
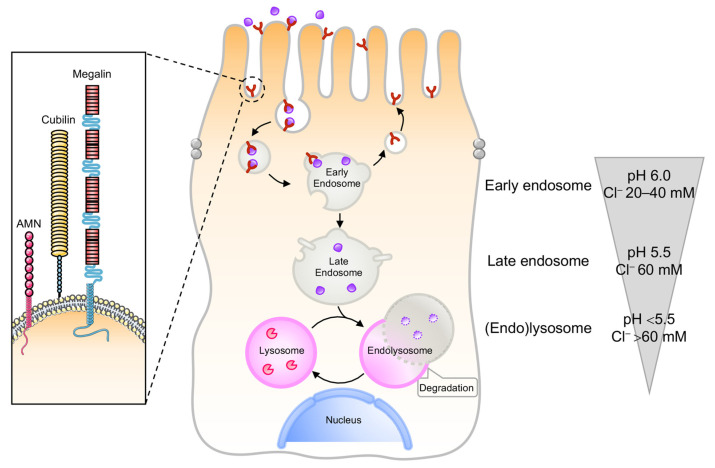
The endolysosome system in the kidney PT. Epithelial cells lining the kidney PT have multifunctional endocytic receptors and a highly developed endolysosome system to take up plasma proteins that are filtered by the glomerulus. The endocytic pathway in PT cells requires coated pits and vesicles, followed by early endosomes that form recycling endosomes or mature to late endosomes that fuse with the lysosome to form the endolysosomes. The luminal pH drops from 7.4 in the tubule lumen to 6.0 in early endosomes, 5.5 in late endosomes, and below 5.0 in endolysosomes. Such vesicular acidification enables the dissociation between receptors and their ligands, the recycling of receptors back to the apical membrane, and the progression of ligands towards the endolysosomal compartments. In parallel, the Cl^−^ concentrations drop from 110 mM in the extracellular space to 20–40 mM in early endosomes, 60 mM in late endosomes, and >80 mM in lysosomes, i.e., much higher than the 10–40 mM in the cytosol.

The kidney PT constitutes a paradigm of effective communication between the environment and endomembrane compartments, guiding the reabsorption of vital nutrients [5,6]. PT cells use receptor-mediated endocytosis and endolysosome-guided transport systems to efficiently reabsorb albumin and low molecular weight proteins (LMWPs) from the ultrafiltrate, preventing the urinary waste of essential proteins under physiological conditions [5,6]. The retrieval of albumin and LMWPs occurs through the multiligand receptors LRP2/megalin and cubilin [11,12,13], and the cooperating protein amnionless (AMN; Figure 1). The binding of filtered ligands to, and the interactions between both endocytic receptors, induces their internalization into clathrin-coated vesicles, and subsequent transport towards the endolysosomal compartments [14,15,16]. An essential component in this trafficking pathway is the apical endosomal compartment, where the ligands opportunely dissociate from their endocytic receptors through a process that requires sustained vesicular acidification (Figure 1) by the electrogenic vacuolar H^+^-ATPase (v-ATPase) proton pump [17,18]. In the kidney PT, additional proteins appear to be involved in the maintenance of the endolysosomal acidification, such as the anion transporter chloride channel 5 and 7 (ClC5 and ClC7; [19]); the cystic fibrosis transmembrane conductance regulator (CFTR; [19]); and the cation transporters mucolipin 1 and two pore calcium channel 1 (TPC1) and TCP2, which mediate Ca^2^^+^ and Na^+^ release from the endolysosome [20]. Once dissociated from their ligands, the endocytic receptors efficiently traffic to subapical Rab11^+^ apical recycling endosomes and successively reach the apical membrane in a microtubule-dependent manner [16], sustaining new cycles of ligand binding and internalization (Figure 1). The generation and maintenance of the endolysosomal pH gradient sustains not only the progression of cargo-filled vesicles towards the endocytic route, but also the activation acid hydrolases within the degradative compartments [21]. Iterative rounds of cargo sorting, coupled with maturation of the early endosomes, result in the formation of late endosomes that fuse with the lysosomes to form endolysosomes (Figure 1), where their accompanying cargoes are eventually degraded [22].

Accumulating evidence suggests that the endolysosome terminates autophagy—an evolutionary conserved pathway that degrades cellular components, such as defective organelles and misfolded proteins, to preserve homeostasis [1,23]. Furthermore, recent studies indicate that autophagy-mediated clearance pathways coordinate the renovation of cells and tissues during kidney development and differentiation, and are also involved in the prevention of genomic damage [24,25]. Therefore, its dysregulation might hasten not only PT dysfunction and kidney disease, but also other pathologies associated with kidney ageing [25,26].

Beyond its role in cellular destruction and quality control, the endolysosome system can steer the metabolic trajectories of cells in response to diverse microenvironmental cues in order to preserve homeostasis [2,4]. Crucial in this process is the (nutrient–dependent) recruitment of the evolutionarily conserved protein kinase called mTOR and its associated regulatory complex 1 (mTORC1) to the surface of the endolysosome through a multiprotein complex [27] comprising Rag guanosine triphosphatases (GTPases) [28,29], Ragulator [30,31], and vacuolar H^+^-adenosine triphosphatase ATPase (v-ATPase) [32]. In the presence of nutrients, the complex localizes on the surface of the endolysosome, where the growth-factor-directed activation of the endolysosome-bound GTPase Rheb [33] allosterically stimulates mTORC1 activity. Signaling from endolysosomes, mTORC1 initiates anabolic programs enhancing growth and proliferation, while suppressing catabolic autophagy and cellular quality control [2,34,35]. In addition, recent studies in rat kidney cells suggest that the reactivation of mTORC1, in combination with the precise regulation of phosphoinositide production, also coordinates autophagic lysosomal reformation (ALR)—an essential process that helps recycle a full complement of functional lysosomes from auto/endolysosomes during prolonged starvation [36]. Furthermore, the cytosolic face of the endolysosome drives the dynamic association of MiT/TFE family basic helix−loop−helix (bHLH) transcription factors, including TFEB, TFE3, TFEC, and MiTF, which that regulate endolysosome biogenesis [34], autophagy [35], and energy metabolism [37], as well as tethering factors that promote endolysosome fusion [2] or contact with other organelles to carry out specific metabolic programs [38]. Intriguingly, the association between mTOR, endolysosome, and the reabsorptive dysfunction in PT cells lacking *Raptor* [39]—the scaffold protein that docks mTOR kinase on the surface of the endolysosome—suggests potential interactions between nutrient sensing, endolysosome-directed mTORC1 signaling, and the maintenance of the kidney PT integrity.

### 2.2. Types of Autophagy

**Figure 2:**
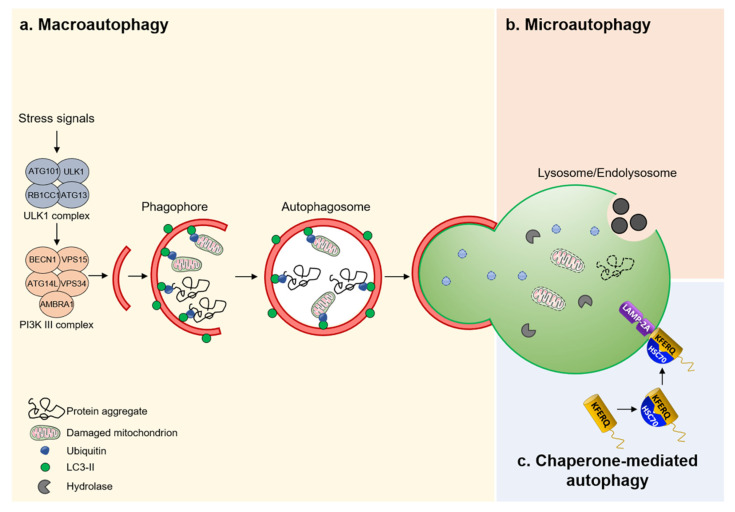
Different mechanisms of autophagy. (**a**) Macroautophagy is a nonselective bulk process that removes protein aggregates and/or damaged mitochondria. (**b**) Microautophagy captures cytoplasmic constituents through direct invagination of endolysosome membranes. Resulting vesicles are subsequently released into the lumen of the endolysosome for degradation. (**c**) CMA identifies proteins containing a KFERQ pentapeptide-related motif by a chaperone complex HSC70 that transports them into the lumen of the endolysosome through a receptor/translocon containing LAMP2A.

Three major routes for the delivery of autophagic cargos to endolysosomes have been reported: macroautophagy, chaperone-mediated autophagy, and microautophagy (Figure 2). Macroautophagy—the best-characterized form of autophagy—involves the sequestration of cellular material within a double-membrane vesicle, termed an autophagosome [40]. Induction factors and stress signals determine the choice of the autophagosome content that can proceed in a relatively nonselective manner, that is the bulk autophagy [41], or entail the tightly regulated disposal of individual cellular components [42]. For instance, mitophagy removes dysfunctional and/or damaged mitochondria; “ribophagy” for ribosomes; “pexophagy” for peroxisomes; “reticulophagy” specifically dismantles portions of the endoplasmic reticulum; ‘‘nucleophagy’’ parts of the nucleus; ‘‘aggrephagy’’selectively removes misfolded protein aggregates, ‘‘lipophagy’’ lipid droplets, and ‘‘xenophagy’’ specifically degrades intracellular bacteria that escape endosomes [42]. Irrespective of substrate specificity, selective autophagy relies on a set of cellular sensors that detect potentially dangerous cues and convert them into signals that are ultimately conveyed to the autophagic machinery [42].

Several distinct complexes containing autophagy-related proteins (ATGs) work with membrane trafficking components to regulate a well-oiled, multistep process that involves initiation, membrane nucleation and phagophore formation, phagophore elongation, cargo sequestration, expansion, autophagosome-lysosome fusion, and degradation. For example, a complex composed of serine/threonine protein kinases ULK1, ULK2, and other proteins stimulates the initiation of autophagy [43,44], while the class III phosphoinositide 3-kinase (PI3K) complex regulates the phagophore formation [45,46]. In addition, two ubiquitin-related systems, i.e., ATG12-ATG5-ATG16L and the microtubule-associated protein 1 light chain 3 (MAP1LC3, also known as LC3), govern the phagophore elongation and sealing of the autophagosome [47]. The autophagy-mediated turnover of damaged and/or dysfunctional mitochondria is required for protecting PT from a wide range of stimuli and insults, such as ischemia, acute kidney injury, sepsis, nutrient deprivation, exposure to toxins and/or pathogens, heat, radiation, hypoxia, and ureteral obstruction [20,48]. Conversely, the deletion of essential autophagy genes (e.g., *Atg5* or *Atg7*; [20,49]) damages PT cells through defective mitochondrial clearance and increased reactive oxygen species (ROS), further substantiating the fundamental role of autophagy in the maintenance of kidney PT integrity and normal physiology. A fascinating interplay between ATG proteins and the membrane dynamics and the nutrient and/or energy-dependent signaling networks that induce autophagy has extensively been described in detail elsewhere [50].

Conversely, chaperone-mediated autophagy (CMA) operates as a protein-exclusive type of autophagy, whereby KFERQ-like motif-bearing proteins are recognized by the heat-shock cognate protein HSPA8/HSC70 and cross the surface of the endolysosome through the binding to lysosomal-associated membrane protein 2A (LAMP2A). This triggers the assembly of receptor/translocon containing LAMP2A that targets the degradation of CMA-flagged substrates by endolysosomes [51]. In contrast to CMA and macroautophagy, microautophagy enwraps, sequesters, and transports cytosolic components into the lumen of endolysosomes without the formation of autophagosomes [52]. The resulting breakdown products generated by the endolysosome-based degradation are eventually exported to the cytoplasm through dedicated nutrient transporters that span the membrane of the endolysosome, and are further utilized for energy or in other metabolic reactions [53]. These recent discoveries are now putting autophagy–endolysosome degradative systems under the spotlight, as they play a key role in safeguarding the homeostasis, integrity, and physiology of the kidney PT.

## 3. Cystinosis as a Paradigm of Endolysosome Disease Causing PT Dysfunction

The dysregulation of the endolysosome system causes a generalized dysfunction of PT cells, ultimately triggering losses of essential nutrients into the urine, thereby causing CKD [54] and life-threatening complications. Such PT dysfunction can stem from rare inherited disorders, owing to the malfunctioning of endolysosome-residing proteins, particularly in cystinosis [5,6].

**Figure 3:**
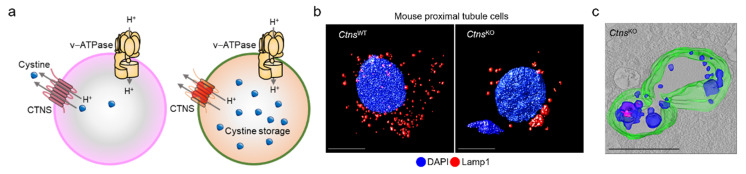
Cystinosin: from endolysosome to disease. (**a**) Cystinosin works in tandem with v-ATPase to transport cystine from the endolysosome, and its absence triggers (**b**,**c**) the storage of cystine within enlarged endolysosomes in primary proximal tubule cells derived from mouse kidneys. Adapted from Festa et al. [62].

Cystinosis—one of a family of approximately 70 rare inborn diseases of the metabolism known as lysosomal storage diseases [55]—is caused by inactivating mutations in the *CTNS* gene encoding the proton-driven transporter cystinosin [56], which exports cystine from the endolysosome (Figure 3a). Given that the low abundance of cystinosin in the lysosomal membrane is the rate-limiting step for cystine transport, its functional loss leads cystine to accumulate within the endolysosomes of tissues across the body, culminating in severe multiorgan dysfunctions that affect primarily the brain, eyes, liver, muscles, pancreas, and kidneys.

The renal Fanconi syndrome is often the first manifestation of cystinosis, usually presenting within the first year of life and characterized by the early and severe dysfunction of PT cells, highlighting the unique vulnerability of kidney cell types [57]. Infantile (MIM #219800) and juvenile (MIM #219900) forms of cystinosis represent a frequent cause of inherited PT dysfunction and renal Fanconi syndrome. In addition, children with cystinosis display early deposition of cystine crystals in the cornea, thereby causing photophobia and painful corneal erosions [58]. In their second to third decade of life, patients with cystinosis can also develop hypothyroidism, hypogonadism, diabetes, myopathy, and deterioration of fine vision and decline of the central nervous system [59,60,61].

The only available strategy to counteract cystine storage is the oral administration of cysteamine, which allows cystine to exit from the endolysosomes [63]. However, cysteamine treatment is hampered by side effects and poor tolerance, and it does not prevent or treat PT dysfunction [63]. Stem cells and gene therapy treatments, which rescued the eyes, kidneys, and thyroid in *Ctns* knockout (KO) mice, and are currently being tested in cystinosis patients, are limited by complexity and high costs [57,64]. Thus, there is an urgent need to identify safe and cost-effective therapeutics for patients with cystinosis. The advent of a growing number of animal and cell-based models that reproduce the human disease pathology has improved our understanding of disease mechanisms and the cellular pathways underlying PT dysfunction and renal Fanconi syndrome, ultimately accelerating the discovery and development of promising new therapeutic approaches. This progress and the recent discoveries are discussed in detail in the next sections.

## 4. Insights into Disease Pathways—The Role of Impaired Autophagy

Recent studies using a *Ctns*^KO^ mouse model that recapitulates multiple features of cystinosis have suggested that the absence of cystinosin in PT cells leads cystine to accumulate within enlarged endolysosomes that move to the perinuclear region and exhibit structural, trafficking, and functional defects (Figure 3b,c). This presumably activates a signaling cascade that drives abnormal cell growth and apical dedifferentiation, ultimately leading to defective receptor-mediated endocytosis and urinary loss of LMW proteins in vivo [65,66]. The tight integration between endolysosome system, regulation of growth signaling pathways, and maintenance of PT differentiation suggests that endolysosome dysfunction driven by cystinosin loss might disrupt the homeostasis in cystinosis-affected PT cells. How, mechanistically, the absence of cystinosin wreaks havoc on cellular homeostasis has remained incompletely understood.

Accumulating evidence suggests that the endolysosome can capture and degrade aged and/or malfunctioning cellular constituents through macroautophagy/autophagy—an evolutionarily conserved, “self–eating” process through which potentially dangerous cytosolic entities are sequestered within autophagosomes and subsequently delivered to endolysosomes for degradation [1,67,68]. This homeostatic process is particularly active in PT cells, whose intense reabsorptive properties require the maintenance of the mitochondrial network [25,69]. Given the structural and functional defects of cystinosis-affected endolysosomes and considering that autophagy relies on catabolic properties of endolysosomes, the cystinosin loss-induced storage of cystine might compromise the degradation of autophagosomes in kidney PT cells. Using differentiated PT cell culture systems, which closely reproduce the key features of the in vivo disease phenotype [62,70] in combination with bona fide autophagy biosensors and assay technologies, Festa and colleagues revealed that primary cells derived from microdissected PT segments of *Ctns*^KO^ (henceforward referred to as mPTCs) mice fail to dismantle Lc3b-flagged autophagosomes [62,71]. Evidence supporting incomplete autophagy flux in cystinosin-deficient PT cells include the following: (i) abnormally high numbers of autophagosomes under-normal growth conditions; (ii) failure to clear autophagic vesicles (AVs) formed after starvation-induced autophagy, mimicking bafilomycin (Bfn) A1 action; (iii) inability of BfnA1 to further elevate the Lc3-II and Sqstm1/p62 protein levels and the numbers of punctate Lc3b-flagged autophagosomes; (iv) and impaired degradation of the resting autophagosomes in *Ctns*^KO^ mPTCs with a selective PI3K3/Vps34 inhibitor [62,71]. Similar autophagy defects (e.g., accumulation of AVs and their defective degradation, and increased p62 levels) have also been observed in several other LSDs (Table 1 [72,73,74,75,76,77,78,79,80,81,82,83,84,85,86,87,88,89,90]). Defects in autophagy–endolysosome degradative pathways, which are also encountered in *ctns*-deficient zebrafish, are reverted by exogenously expressing wild-type cystinosin in mutant cells [62,71]. Of note, treatment with the oral drug cysteamine, which efficiently depletes the storage of cystine within endolysosomes, does not restore the functioning of endolysosomes nor the catabolic autophagy in patient cells [91,92]. Thus, cystinosin—beyond its function in cystine transport—might act as an evolutionarily conserved, metabolic rheostat that regulates the response of endolysosomes to the arrival of endocytosed and autophagy cargoes, hence safeguarding the integrity and the physiological homeostasis of kidney tubule cells [62,71].

Conversely, Napolitano and colleagues indicate that the macroautophagy/autophagy flux seems to be fully normal, despite the increased number of autophagosomes in mutant cells [93]. Thus, it is plausible that elevated numbers of autophagosome could stem from compensatory mechanisms due to defects in CMA. Indeed, studies in cultured cells (e.g., hepatocytes and T cells) have indicated a functional crosstalk between macroautophagy and CMA, whereby cells respond to the failure of one of these pathways by activating the other [94,95]. In line with this concept, studies on lysosomes purified from livers of starved *Ctns*^KO^ mice unveil defects in the degradation of glyceraldehyde-3-posphate dehydrogenase (GADPH)—a well-established substrate for the CMA pathway [51]. These abnormalities are reflected by dislodgement of the lysosomal receptor Lamp2a required for CMA from its natural binding partner Lamp1 and co-localization with Rab11a-positive recycling endosomes. These trafficking defects appear to be specific for Lamp2a, as other Lamp proteins can normally reach the endolysosomes in cystinosis-affected fibroblasts [93]. The small GTPase Ras-related protein Rab-11A (RAB11), and the RAB7 effector Rab-interacting lysosomal protein (RILP), seem to be part of this trafficking machinery, as the correction of the lower levels observed for both proteins in patient cells is sufficient to repair LAMP2A mistargeting, and hence the CMA pathway in diseased cells [96]. Such an apparent discrepancy might be attributed to: (i) differential biochemistry of distinct cell types in the body; (ii) turnover rates of autophagy cargos and substrates; (iii) cell type and tissue/organ-dependent adaptative responses to counteract the primary storage defect, and (iv) whether the cells are renewing or terminally differentiated; and (v) differential threshold triggered by cystine storage to induce dysfunction in distinct cell types, and affected tissues and organs. However, in some studies, exogenous expression of the dynein subunit, e.g., DYNC1LI2 (dynein, cytoplasmic 1 light intermediate chain 2)—a key cytoskeletal motor protein involved intracellular transport of cargo, organelle trafficking, mitotic spindle assembly, and positioning—rescues the localization of the chaperone-mediated autophagy (CMA) receptor LAMP2A, CMA activity, and the cellular homeostasis in cystinosis-affected PT cells [97]. Regardless of the mechanisms involved, the concept that defects in endolysosomes and autophagy pathways might contribute to cystinosis pathogenesis is in line with recent studies that indicate an accumulation of autophagosomes engulfing damaged and/or dysfunction mitochondria, and increased formation of aggregate-prone SQSTM1/p62 inclusions in both kidney biopsies [98,99] and patient cells [100].

## 5. Autophagy, Mitochondria, and Epithelial Dysfunction in Cystinosis

The conjugation of defective endolysosome dynamics and impaired catabolic properties is strikingly similar to cellular alterations stemming from the accumulation of monoclonal light chains (κLCs) within the endolysosomes of PT cells, causing a similar epithelial dysfunction [7]. Furthermore, the uncontrolled increase in the endolysosomal PtdIns(4,5)P_2_ pool that arises from loss-of-function of the PtdIns(4,5)P_2_ 5-phosphatase OCRL triggers endolysosome dysfunction and autophagosome accumulation of patients with Lowe syndrome [102,103]—another rare inherited disorders causing PT dysfunction and renal Fanconi syndrome. The storage of either cystine or κLCs or PtdIns(4,5)P_2_ might thus tamp down the homeostasis and transport functions of PT cells, emphasizing the crucial role of autophagy–endolysosome degradative systems in preserving the homeostasis and physiology of the kidney PT.

As a direct consequence of defective autophagy–endolysosome degradation systems, *Ctns*^KO^ PT cells remarkably accumulate SQSTM1- and ubiquitin-forming aggregates with damaged and/or dysfunctional mitochondria within enlarged, non-degradative endolysosomes, ultimately overproducing mitochondrial-derived reactive oxygen species (ROS) [62,71]. Genetic (e.g., short hairpin RNA interference targeting *Atg7*) and pharmacological (e.g., inhibition of Beclin1/Vps-34 complex by using SAR-405 or Spautin-1) suppression of autophagy dampens the functioning of the mitochondrial network, inducing oxidative stress, while repressing the receptor-mediated endocytosis and transport properties of PT cells [62,71]. This evidence further reinforces the mechanistic connection between defective mitochondrial quality control, oxidative stress, and cellular dysfunction. Thus, the maintenance of degradative autophagy might serve as a bona fide (homeostasis-modifying) process that regulates the identity of the kidney tubule cells. How, mechanistically, defects in degradative autophagy disrupting the differentiation of PT cells have remained to be fully elucidated.

**Figure 4:**
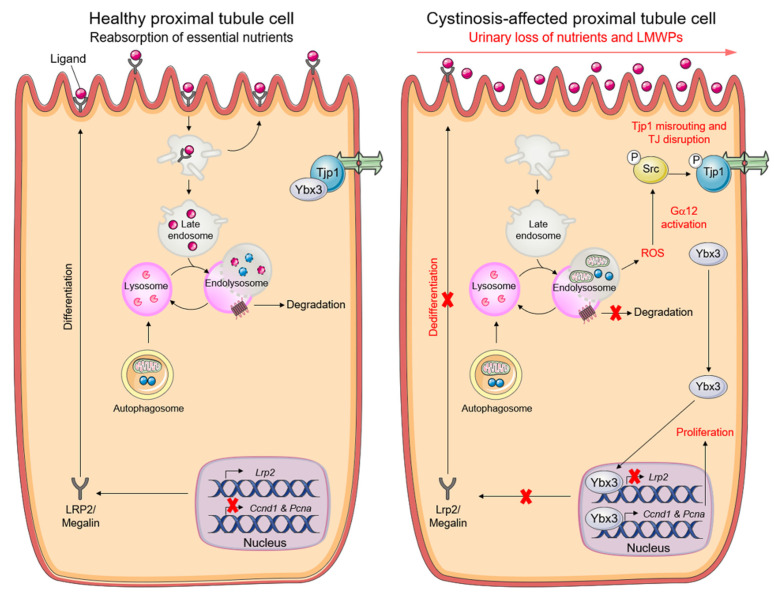
Pathogenic cascade driving PT dysfunction in cystinosis cells. Graphical schematic illustrating that cystinosin-deficient PT cells accumulate dysfunctional mitochondria and reactive-oxygen species (ROS), triggering an abnormal activation of the tight junction—associated signalling that stimulates proliferation while suppressing apical dedifferentiation. Reprinted with permission from Ref. [6]. Copyright 2021 Springer Nature.

Recent insights have illuminated the biological functions of tight junction proteins in safeguarding the epithelial cell behavior and phenotype. In particular, tight junction adaptor protein 1 (Tjp1) represses the nuclear translocation of an Y box binding protein 3 (Ybx3) —a transcriptional factor that promotes cell proliferation while repressing PT differentiation during kidney development [104]. As oxidative stress damages tight junction integrity [105], the excessive mitochondrial ROS induced by cystinosin loss might trigger an abnormal activation of the tight junction associated Ybx3 (Y box binding protein) signaling, which would, in turn, lead to epithelial dysfunction in cystinosis PT cells. In line with this model, increased levels of mitochondrial ROS stimulate Gna12/Ga12-SRC-mediated phosphorylation of Tjp1 and its subsequent misrouting to enlarged, non-degradative endolysosomes. The disruption of tight junction integrity triggers the hyperactivation of tight junction-associated Ybx3 signaling, with increased proliferation (e.g., *Ccnd1* and *Pcna*) and reduced apical differentiation (e.g., *Lrp2*), ultimately disabling receptor-mediated endocytosis and epithelial functions in *Ctns*^KO^ cells [62,71] (Figure 4). Gain- and loss-of-function approaches targeting *Gna12* or *Tjp1* or *Ybx3*, or pharmacological interventions impeding activation of the Gna12-Src-directed signaling (e.g., with the mitochondrial-targeted antioxidant Mito-TEMPO or with the SRC inhibitor SU6656) restore epithelial functions in *Ctns*^KO^ cells [62,71]. By regulating autophagy and the Tjp1-Yxb3 signaling, the crosstalk between cystinosin and the endolysosome system might thus dictate the balance between proliferation and differentiation of PT cells, and hence their role in homeostasis.

## 6. Pharmacological Modulation of Autophagy as a Targetable Pathway in Cystinosis

There are no curative treatments for cystinosis, and the current supportive care approaches have substantially decreased mortality and overall morbidity. For example, supplementation with water, bicarbonate, citrate, phosphate, salts, and vitamin D can rapidly attenuate the metabolic complications associated with renal Fanconi syndrome, and hence maintain an adequate body fluid and electrolyte homeostasis [6,57]. Beyond management care, patients with cystinosis can benefit from treatment with cysteamine [58]—an FDA-approved drug that depletes the endolysosomal cystine storage by cleaving cystine into free cysteine and cysteamine–cysteine mixed sulphide. These metabolites are subsequently exported from the endolysosome to cytoplasm through cationic amino acid transporter 2 (PQLC2), which spans the endolysosomal membrane [106]. Despite an improvement in patients’ quality of life, treatment is hampered by adverse effects, poor tolerance, and a strict dosing schedule, and it does not prevent or treat the renal Fanconi syndrome and kidney failure [58,63]. Therefore, there is an urgent need to yield promising new targetable interventions in the early course of cystinosis.

**Figure 5:**
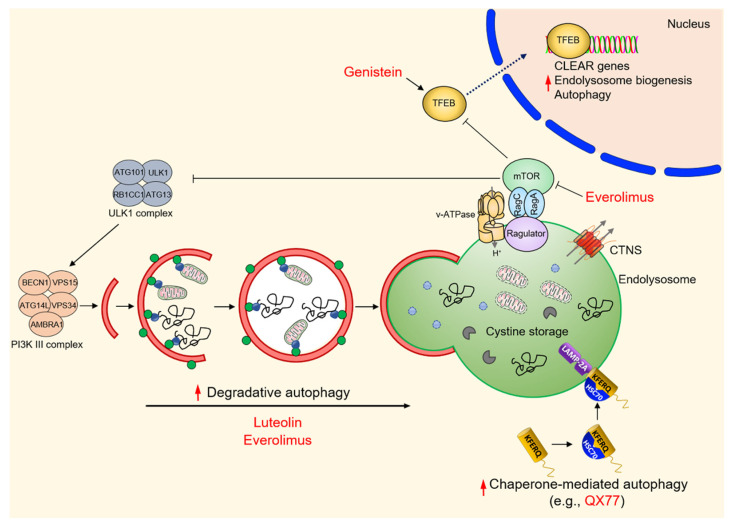
Summary of emerging therapeutic strategies targeting autophagy pathways in cystinosis.

The molecular understanding of regulatory circuitries coupling endolysosome disease, autophagy, and epithelial dysfunction might thus guide the discovery and development of targeted therapeutics for cystinosis patients [57,64]. In this case, interventions that are aimed to target each step of the pathogenic cascade might mediate beneficial effects and potentially counteract the homeostatic perturbations imposed by cystinosin loss and the resulting cystine storage. For example, small molecule compounds that either activate CMA [107] or boost the excretion [108] of cystine-loaded endolysosomes might ameliorate clinical outcomes if they are used concomitantly with the cystine-depleting drug cysteamine [93,109] (Figure 5). Boosting CMA with small-molecule activators (e.g., QX77) increases the lifetime of the endocytic receptor megalin at the plasma membrane, ultimately improving the epithelial functions in human PT cells lacking cystinosin (e.g., CRISPR-Cas9-induced gene deletion) [101]. Consistent with these observations, combinatorial strategies using an mTORC1 inhibitor (e.g., everolimus) and cysteamine rescues the homeostasis and functioning of autophagy–endolysosome degradation systems in cystinosis patient-derived pluripotent stem cells (iPSCs) and kidney organoid models of the disease [110] (Figure 5).

Despite normal mTORC1 activity in cystinotic iPSCs and their derived kidney organoids, the molecular mechanisms behind the beneficial effects of the combo treatment remain largely elusive. In this setting, a potential mediator could be the activation of TFEB—a master regulator that controls the expression of the genes involved in autophagy and endolysosome biogenesis [2,34,35] (Figure 5). Recent work showing that cystinosin might physically interact with many components of the v-ATPase–Ragulator–Rag complex [110,111], which regulates the mTORC1 lifetime and its activation at the surface of the endolysosome, and that the reconstitution of TFEB signaling stimulates the catabolic properties of endolysosomes and the completion of autophagy in conditionally immortalized PT epithelial cells (ciPTEC) derived from the urine of a cystinotic patient [92], further substantiate the concept. Indeed, the pharmacological induction (e.g., genistein) of nuclear translocation and the activation of TFEB-dependent transcriptional programs has recently been shown to empty cystine storage, to restore the functioning of endolysosomes and degradative autophagy, and to improve the processing of endocytosed cargos in cystinotic ciPTEC [92] (Figure 5).

Recently, drug discovery and repurposing strategies are gaining momentum as a default tool for providing affordable therapies in rare inherited diseases [112,113]. With the possibility to screen approved and investigational products, the process is well adapted to the curiosity-driven research culture in academia, hence mitigating the risk inherent in preclinical drug discovery. With this lag in mind, De Leo and colleagues recently identified small molecule drug candidates [100] that decrease the accumulation of the autophagy substrate p62/SQSTM1 and restore the autophagy–endolysosome degradative pathways, which are compromised in different models and cell systems of cystinosis [100]. Among several positive hits, luteolin—a natural flavonoid that is present in various fruits and vegetables—has emerged as the most interesting candidate. This compound has a good safety profile, owing to its similarity to genistein, and improves the endolysosome-mediated degradation of autophagy cargoes and substrates, including damaged and dysfunctional (ROS-overproducing) mitochondria [100] (Figure 5). In addition, treating cystinotic ciPTEC, mPTCs derived from mouse *Ctns*^KO^ kidneys, and zebrafish models of cystinosis with luteolin not only repaired endolysosomes, autophagy degradation, and mitochondrial redox homeostasis and cellular distress, but also restored megalin expression at the plasma membrane, ultimately stimulating protein absorption and hence transport functions [100]. These findings extend previous observations demonstrating that structural and functional deformities of the kidney PT could be delayed in *Ctns*^KO^ mice by administrating mitochondria-targeted ROS scavengers such as mitoquinone [114] or mito-TEMPO [62]. Thus, the modulation of autophagy–endolysosome degradative systems might offer a promising new therapeutic avenue not only for cystinosis, but also for other currently intractable diseases related to endolysosome storage.

## 7. Concluding Remarks

The maintenance of a healthy endolysosomal system is particularly crucial for preserving the homeostasis and physiology of kidney tubule cells, and loss-of-function mutations that impair the functioning of the endolysosome system can invariably lead to PT dysfunction and kidney disease. Rare inherited defects in an endolysosome-residing protein and the storage materials, as exemplified here by cystinosis, might disable autophagy and organelle quality control, triggering a level of mitochondrial distress that drives the dysfunction of the kidney PT. Further studies will be required for understanding whether other LSDs might have various degrees of PT dysfunction and kidney disease. In most cases, kidney disease manifestations might be overshadowed by more severe symptoms affecting the brain, underestimating the prevalence of kidney involvement in these disorders.

The mechanisms by which cystinosin deficiency wreaks havoc on homeostasis and function of the endolysosome system remain largely elusive. These defects could stem from defects in mannose-6-phosphate (M6P)-dependent trafficking [115] or megalin-directed reuptake of filtered lysosomal cathepsins [116] or endolysosome acidification [117]. Alternatively, the storage of cystine wrought by cystinosin loss might affect the folding of disulphide-bonded substrates for endoproteolytic attack or thiol-active catalytic sites of endolysosomal cathepsins, ultimately affecting processing and their lasting maturation [57,118].

As the endolysosome is the site for nutrient sensing and the activation of mTORC1 signaling—the master regulator that represses autophagy and endolysosome biogenesis—it will be important to evaluate whether cystinosin deficiency and cystine storage might contribute to hyperactive mTORC1. This might in turn inhibit endolysosome and autophagosome biogenesis, thus generating a vicious cycle that boosts metabolic dyshomeostasis and dysfunction in cystinosis cells. Although cystinosin could physically interact with many components of the v-ATPase–Ragulator–Rag complex [111] that regulates mTORC1 activity, the contribution of dysregulated nutrient sensing and mTOR signaling to disease pathogenesis remains an open question. The recent development of model organisms [62] and primary PT cell culture systems [65,70], which closely reproduce the key features of the disease phenotype, and mass spectrometry-based profiling of intact endolysosomes [119], presents an opportunity to address this critical point.

Decline in endolysosome function and mitochondrial autophagy are clear hallmarks of ageing, and correlate with metabolic dysfunction [3]. Indeed, the behavior of “aged” endolysosomes mimics the cellular phenotypes encountered in cystinosis and other LSD cells. We suspect that the dysregulation of adaptive response to mitochondrial distress might also contribute to maladaptation and disease in patients with cystinosis, and this will require further studies to understand the effects of *CTNS* mutations on organelle repair pathways, such as mitochondrial unfolded protein response (UPRmt) and mitochondrial biogenesis. The increasing power of organelle-specific purification and profiling via proteomic, lipidomic, and metabolomic-based approaches will be useful in filling these missing knowledge gaps. These questions are just examples of all the exciting work that lies ahead to comprehensively dissect the biological functions of cystinosin in the context of tissue homeostasis and disease.

A current challenge is to translate the knowledge gained from fundamental studies of endolysosome biology to the treatment of cystinosis and other endolysosome-related diseases. In this regard, the use of informative preclinical models, coupled with improved knowledge of disease signatures and the recent advances in multi-omics technologies, might accelerate the discovery and development of “first-in-class” therapeutics that can halt the progression of cystinosis, as well as other rare and more common diseases related to endolysosome dysfunction.

## Figures and Tables

**Table 1:** Autophagy pathways in lysosomal storage diseases.

Mechanisms of Lysosomal Storage	Disease Examples	Lysosomal Protein Defect	Substrate	AV Accumulation	Defective AV Degradation	CMA Activity	Increased SQSTM1/p62	Refs.
Lysosomal enzyme deficiencies	Fabry	α-Galactosidase	(Lyso-) Globotriaosylceramide	Yes	Yes	??	Yes	[81]
	Gaucher	β-Glucocerebrosidase	Glucosylceramide, glucosylsphingosine	Yes	Yes	??	Yes	[80,85,86]
	Mucopolysaccharidoses	Enzymes involved in Mucopolysaccharide catabolism	Mucopolysaccharides	Yes	Yes	??	Yes	[75,76,77]
	Multiple sulfatase deficiency	SUMF1 (Activator of sulfatases)	Multiple, including sulfated glycosaminoglycans	Yes	Yes	??	Yes	[75,76]
	Pompe	α-Glucosidase	Glycogen	Yes	Yes	??	Yes	[72,73,87,88]
Defects in soluble non−enzymatic lysosomal proteins	Niemann-Pick disease type C2	NPC2	Cholesterol and sphingolipids	Yes	Yes	??	Yes	[78,79]
Defects in lysosomal membrane proteins	Cystinosis	Cystinosin	Cystine	Yes	Yes	Reduced	Yes	[62,71,93,97,100,101]
	Danon	Lysosomal-associated membrane protein 2, splicing variant A (LAMP2)	Glycogen and other autophagic components	Yes	Yes	??	Yes	[74]
	Mucolipidosis IV	Mucolipin-I	Mucopolysaccharides and lipids	Yes	Yes	Reduced	Yes	[82,83,84]
	Niemann-Pick disease type C1	NPC1	Cholesterol and sphingolipids	Yes	Yes	??	Yes	[78,79,88]
